# Trends in the prevalence and incidence of Crohn’s disease in Japan and the United States

**DOI:** 10.1007/s00384-024-04636-5

**Published:** 2024-04-27

**Authors:** Ko Nakajo, Michiyo Yamazaki, Hsingwen Chung, Youran Xu, Hong Qiu

**Affiliations:** 1 Global Epidemiology, Office of the Chief Medical Officer, Janssen Research & Development, LLC, Tokyo, Japan; 2 Global Epidemiology, Office of the Chief Medical Officer, Janssen Research & Development, LLC, Titusville, USA; 3 Global Epidemiology, Office of the Chief Medical Officer, Janssen Research & Development, LLC, Beijing, China

**Keywords:** Epidemiology, Crohn’s disease, Incidence, Prevalence, Japan, United States

## Abstract

**Purpose:**

This study is to describe patient demographic characteristics and estimate annual prevalence and incidence rates of Crohn’s disease (CD) in Japan and the United States (US).

**Methods:**

Two large employment-based healthcare claims databases (Japan Medical Data Center [JMDC] in Japan and Merative MarketScan [Merative] in the US) were used to identify patients with CD from 2010 to 2019. Cases were confirmed using an algorithm based on diagnostic with/without treatment codes. The Merative population was used for sex and age standardization of annual prevalence and incidence rates estimated from the JMDC.

**Results:**

Patients with CD were generally younger in Japan than in the US at diagnosis (mean 33.6 vs. 39.4 years) and 71.5% were male versus 45.1% in the US. Annual prevalence per 100,000 population increased substantially in both countries, from 34.2 in 2010 to 54.5 in 2019 in Japan (standardized) and 163.3 to 224.2 in the US. Prevalence rates increased in both males and females in all age groups between 6 and < 65 years. Annual incidence rate per 100,000 person-years was almost fourfold higher in the US than Japan (21.0 vs. 5.5 [standardized] in 2019) but remained stable in both countries over time in both sexes and in all age groups.

**Conclusion:**

The epidemiology of CD differs between Japan and the US. Research to understand the basis of these differences could help to identify at-risk groups in each country, and guide implementation of preventive measures.

**Supplementary Information:**

The online version contains supplementary material available at 10.1007/s00384-024-04636-5.

## Background

Crohn’s disease (CD) is a chronic, progressive, inflammatory bowel disease (IBD) that can affect any segment of the gastrointestinal tract but usually involves the ileocecal region. Patients typically present with diarrhea (with or without blood), abdominal pain, and weight loss. At diagnosis, around one-third of patients have ileitis, colitis or ileocolitis, and one-third have intestinal complications such as stricture or penetrating disease [1, 2]. Other complications include anal fissures, abscesses, and peritonitis, and up to 70–80% of patients will require surgical intervention within 20 years [2]. CD typically waxes and wanes, and while most patients have a relapsing and remitting disease course, 13% experience unremitting disease [1]. In Western countries, the peak age of onset is between 20 and 30 years and women are affected more often than men [3].

IBD was previously considered to be a disease of Western societies but in the twenty-first century, the incidence and prevalence of IBD have increased in Asia, South America, and Africa [4, 5]. In North America, reported incidence rates of CD range from 6.30 to 23.82 per 100,000 person-years, and in Eastern Asia from 0.06 to 3.32 per 100,000 person-years [4]. The prevalence of CD in Japan has steadily increased since the 1970s [6]. While still lower than in Western countries, the nationwide registration system of intractable diseases in Japan recorded an increase in the prevalence of CD from 5.9 to 21.1 per 100,000 population between 1991 and 2005 [7], and estimated prevalence to be 30.1 per 100,000 population in 2013 [8]. There were 70,700 persons estimated to have CD in 2014, two-thirds of them male [9].

In the United States (US), the prevalence of CD approximately doubled between 2007 and 2016 [10]. The incidence of CD in a study conducted in Olmsted County, Minnesota increased from the 1970s until 2010 and was reported as 10.7 per 100,000 person-years between 2000 and 2010 [11]. By contrast, a national database study in US veterans reported stable incidence rates between 1997 and 2009 that ranged between 26 and 40 per 100,000 person-years [12]. Contemporary national data describing the epidemiology of CD in the US are lacking.

The epidemiology of CD appears to differ between Western and Asian countries in terms of age, sex distribution, and clinical course, but has not been well described [8]. Geographic variability in polymorphisms that influence the risk of IBD may contribute to some of these differences [13]. We recently reported the results of a retrospective cohort study to estimate the annual prevalence, incidence, and demographic characteristics of patients with ulcerative colitis (UC) in Japan and the US over a 10-year period from 2010 to 2019 [14]. In this parallel study, we used the same databases to estimate annual prevalence and incidence rates, and to describe demographic characteristics of patients with CD in Japan and the US.

## Methods

### Study design and data sources

The data sources used in this analysis have been described previously [14]. In brief, the Japan Medical Data Center (JMDC) is an employment health insurance claims database that commenced in 2005. It captures inpatient, outpatient, and pharmaceutical healthcare services from more than 250 payers nationally [15]. The JMDC holds data from approximately 14 million non-government employees aged 18 to 65 years and their dependents (children < 18 years of age and adults aged < 75 years) [16]. Claims data are derived from monthly claims issued by clinics, hospitals, and pharmacies. Diagnoses are coded in International Classification of Disease 10 (ICD-10) format and medications in Anatomical Therapeutic Chemical format. Data collection ceases if members become unemployed or not fit to work.

The Merative™ MarketScan^®^ (“Merative” previously IBM MarketScan Commercial Claims and Encounters) database includes health insurance claims from inpatient, outpatient, outpatient pharmacy, and behavioral healthcare episodes from more than 155 million employees aged up to 65 years across the US [17]. Diagnoses are coded in ICD-9 and ICD-10 formats.

All data were de-identified and fully compliant with relevant patient confidentiality requirements, including the Japan Act of the Personal Information Protection and the Health Insurance Portability and Accountability Act of 1996 in the US. Ethical approval and individual informed consent were not required.

### Study population and case identification

All individuals in the databases aged < 65 years were eligible for the study if they had been continuously enrolled in the health insurance plan for at least 12 months at any given time during the study period from 2010 to 2019.

The study population and case identification strategy used the previously reported case identification algorithm using diagnostic with/without treatment codes [14]. A diagnosis of IBD was considered confirmed if a patient had ≥ 2 IBD-related healthcare encounters on different days with an ICD-9 diagnosis code 555.x (CD), 556.x (UC), or ICD-10 diagnosis code K50.x (CD) or K51.x (UC) [18]. Patients with a single IBD diagnosis were also considered as confirmed cases if they had at least one pharmacy claim for an IBD-specific medication on the day of, or ± 1-year from the IBD diagnosis code. Patients were followed until they left the health plan or until study end (December 31, 2019), whichever occurred first.

### Overall cohort (2010–2019)

There were three study cohorts: the overall cohorts comprised the overall General Population cohort, which included any eligible patient in the databases during the study period; the overall Prevalent CD cohort which included patients in the overall General Population cohort with confirmed CD; and the overall Incident CD cohort which included patients in the overall Prevalent CD cohort with at least 12 months of continuous enrollment prior to the index date, and no diagnosis or pharmacy CD code prior to the index date from the patients’ first observation in the databases, starting from year 2005 for JMDC and 2000 for Merative.

### Study cohorts

Each overall cohort was stratified by calendar year into ten sub-cohorts; the calendar-year General Population cohort; the calendar-year Prevalent CD cohort; and the calendar-year Incident CD cohort. The calendar-year cohorts were used as denominators and numerators for estimations of annual prevalence and annual incidence for the equivalent calendar year.

### Variables

The index date was the date of the first qualifying CD code (diagnostic or pharmacy) either over the study period for the overall Prevalent and Incident CD cohorts, or in the equivalent calendar year for the calendar-year Prevalent and Incident CD cohorts. Baseline demographic characteristics of patients were measured on the index dates. The year of the first qualifying diagnosis and pharmacy codes for CD in the overall Prevalent and Incident cohorts were recorded.

### Statistical analyses

Continuous variables measured at cohort entry were presented descriptively. Frequencies and percentages were reported for categorial variables including sex (male, female), age (< 6, 6 to < 18, 18 to < 45, and 45 to < 65 years), year of the first qualifying diagnosis and pharmacy codes.

Period prevalence rates per 100,000 population and period incidence rates per 100,000 person-years were estimated annually in each database using the respective calendar-year Prevalent/Incident CD cohorts. The risk period for any patient in the calendar-year Incident CD cohort was from January 1 until the index date. Person-years of incident CD cases that occurred in the preceding years were removed.

In the Prevalent CD cohort, denominators to estimate age-group specific and sex-specific prevalence were the total number of persons in the calendar-year General Population cohort in each relevant age group strata and sex strata. In the Incident CD cohort, denominators used to estimate age group–specific and sex-specific incidence rates were the total person-years of the at-risk population in each relevant age group and sex-specific strata in the equivalent year.

The age distribution (stratified by sex) of the Merative population was used as the standard population for each calendar year and crude period prevalence and incidence rates estimated in the JMDC were directly standardized against the Merative population.

All analyses were performed using Aginity Workbench for Amazon Redshift (Version 4.9.3.2778) and SAS Enterprise Guide 7.15.

## Results

### Study population

**Fig. 1:**
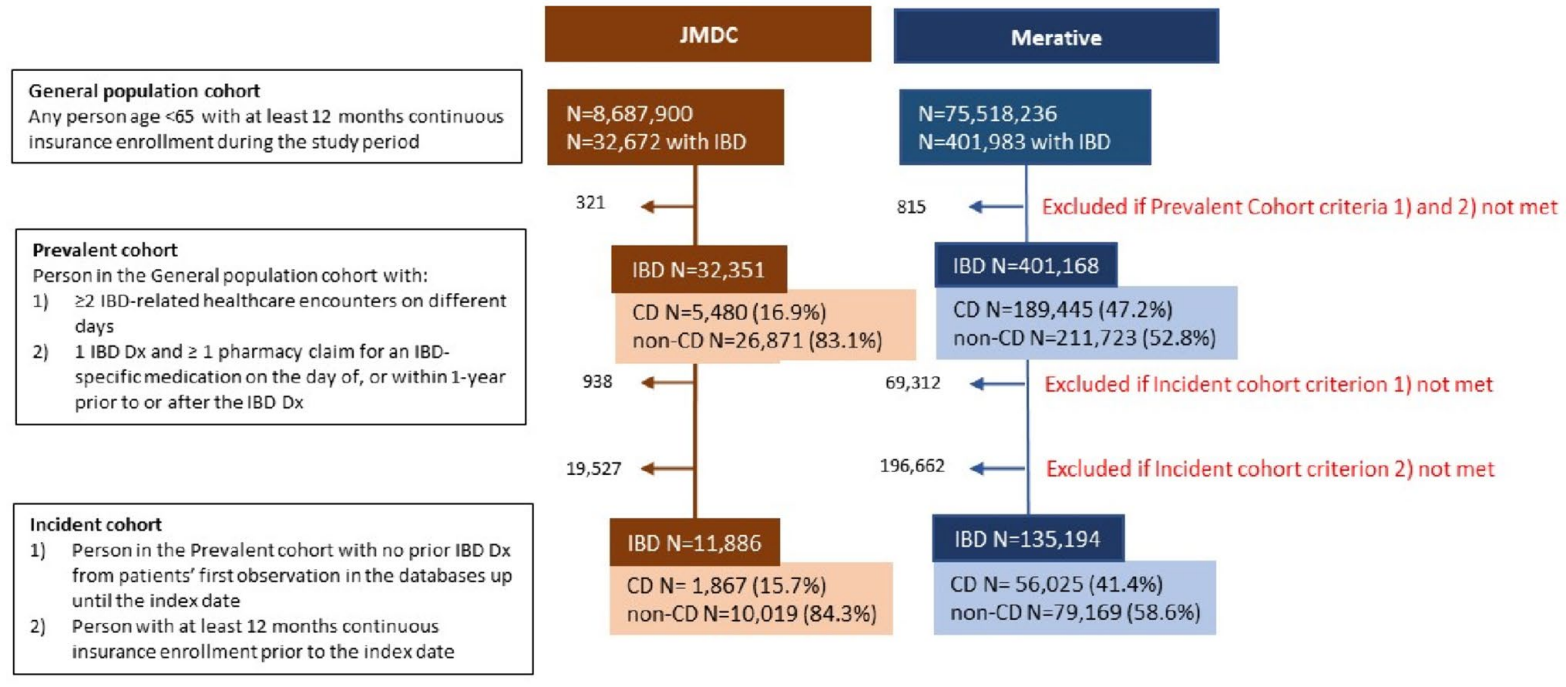
Patient entry into study cohorts in the JMDC (Japan) and Merative (US) Databases (January 1, 2010 to December 31, 2019). Merative, Merative™ MarketScan^®^; Dx, diagnosis; IBD, inflammatory bowel disease; JMDC, Japan Medical Data Center; N, number of patients; CD, Crohn’s disease

The General Population cohort in JMDC (Japan) increased 7.2-fold from 2010 to 2019, while in Merative (US) it increased 1.2-fold from 2010 to 2012 and decreased by 0.58-fold thereafter [14]. Among 32,351 unique cases of IBD in the JMDC, 5480 (16.9%) had a confirmed diagnosis of CD. Among 401,168 unique cases of IBD in Merative, 189,445 (47.2%) had a confirmed diagnosis of CD. The incident CD cohort comprised 1867 patients in JMDC and 56,025 in Merative (Fig. 1).

Within each database, the median age of patients was similar in the overall Prevalent and Incident cohorts, although the median age was lower in JMDC (36.1 and 31.9 years, respectively in the overall Prevalent and Incident cohorts) than in Merative (41 years in both cohorts) (Table 1). Within the overall Prevalent and Incident cohorts in JMDC, 73.0% and 74.9%, respectively were aged < 45 years of age at the index date, versus 57.4% and 56.8%, respectively in Merative. Males outnumbered females in the JMDC (75.80% in the overall Prevalent cohort and 71.5% in the overall Incident cohort) while 45.1% and 45.3%, in the respective cohorts were male in Merative.

**Table 1:** Characteristics of patients with Crohn’s disease at the index date in the overall Prevalent and Incident CD cohorts in the JMDC (Japan) and Merative (US) Databases (January 2010 to December 2019)

	**Merative**		**JMDC**	
**Characteristic**	**Incident cohort**	**Prevalent cohort**	**Incident cohort**	**Prevalent cohort**
	**(*N* = 56,025)**	**(*N* = 189,445)**	**(*N* = 1,867)**	**(*N* = 5,480)**
**Age, years**				
Mean (SD)	39.4 (15.6)	40.1 (14.6)	33.6 (14.4)	36.1 (12.7)
Median	41	41	31.9	36.1
Min–max	1–64	1–64	1–64.9	0.2–64.9
Q1–Q3	26–53	28–53	21.4–45	25.8–45.8
**Age group, *n* (%)**				
< 6	212 (0.4)	384 (0.2)	15 (0.8)	24 (0.4)
6 to < 18	5704 (10.2)	13,060 (6.9)	261 (14)	346 (6.3)
18 to < 45	25,900 (46.2)	95,200 (50.3)	1122 (60.1)	3632 (66.3)
45 to < 65	24,209 (43.2)	80,801 (42.7)	469 (25.1)	1478 (27)
**Sex, *n* (%)**				
Female	30,660 (54.7)	103,988 (54.9)	533 (28.5)	1325 (24.2)
Male	25,365 (45.3)	85,457 (45.1)	1334 (71.5)	4155 (75.8)
**Year of first qualifying diagnostic code, *n* (%)**				
2010	5789 (10.3)	47,487 (25.1)	33 (1.8)	390 (7.1)
2011	7160 (12.8)	25,824 (13.6)	61 (3.3)	175 (3.2)
2012	7863 (14.0)	20,658 (10.9)	79 (4.2)	275 (5.0)
2013	6018 (10.7)	19,598 (10.3)	96 (5.1)	558 (10.2)
2014	6626 (11.8)	16,132 (8.5)	151 (8.1)	252 (4.6)
2015	5286 (9.4)	12,549 (6.6)	167 (8.9)	808 (14.7)
2016	4975 (8.9)	12,350 (6.5)	246 (13.2)	918 (16.8)
2017	4277 (7.6)	12,326 (6.5)	299 (16.0)	880 (16.1)
2018	4130 (7.4)	12,873 (6.8)	357 (19.1)	926 (16.9)
2019	3901 (7.0)	9648 (5.1)	378 (20.2)	298 (5.4)
**Year of first qualifying pharmacy code**^a^**, *n* (%)**				
2010	4492 (8)	18,292 (9.7)	20 (1.1)	171 (3.1)
2011	5321 (9.5)	18,841 (9.9)	45 (2.4)	156 (2.8)
2012	5846 (10.4)	17,085 (9)	60 (3.2)	242 (4.4)
2013	4918 (8.8)	16,444 (8.7)	73 (3.9)	502 (9.2)
2014	4921 (8.8)	13,560 (7.2)	113 (6.1)	228 (4.2)
2015	4190 (7.5)	11,024 (5.8)	133 (7.1)	719 (13.1)
2016	4217 (7.5)	11,136 (5.9)	188 (10.1)	854 (15.6)
2017	3575 (6.4)	10,872 (5.7)	222 (11.9)	811 (14.8)
2018	3484 (6.2)	11,483 (6.1)	310 (16.6)	900 (16.4)
2019	3088 (5.5)	8694 (4.6)	297 (15.9)	294 (5.4)
Total	44,052 (78.6)	137,431 (72.5)	1461 (78.3)	4877 (89.0)

*Merative* Merative™ MarketScan^®^, *JMDC* The Japan Medical Data Center, *N* number of patients, *Q1–Q3* interquartile range, *SD* standard deviation

^a^Pharmacy code used for case identification included CD-specific medication

The number of incident cases of CD in the JMDC increased annually from 33 (1.8% of all cases) in 2010 to 378 (20.2%) in 2019, whereas the number of incident CD cases in Merative peaked in 2012 at 7863 (14%) and declined thereafter to 3901 (7.0%) in 2019.

### Period prevalence of CD

**Fig. 2:**
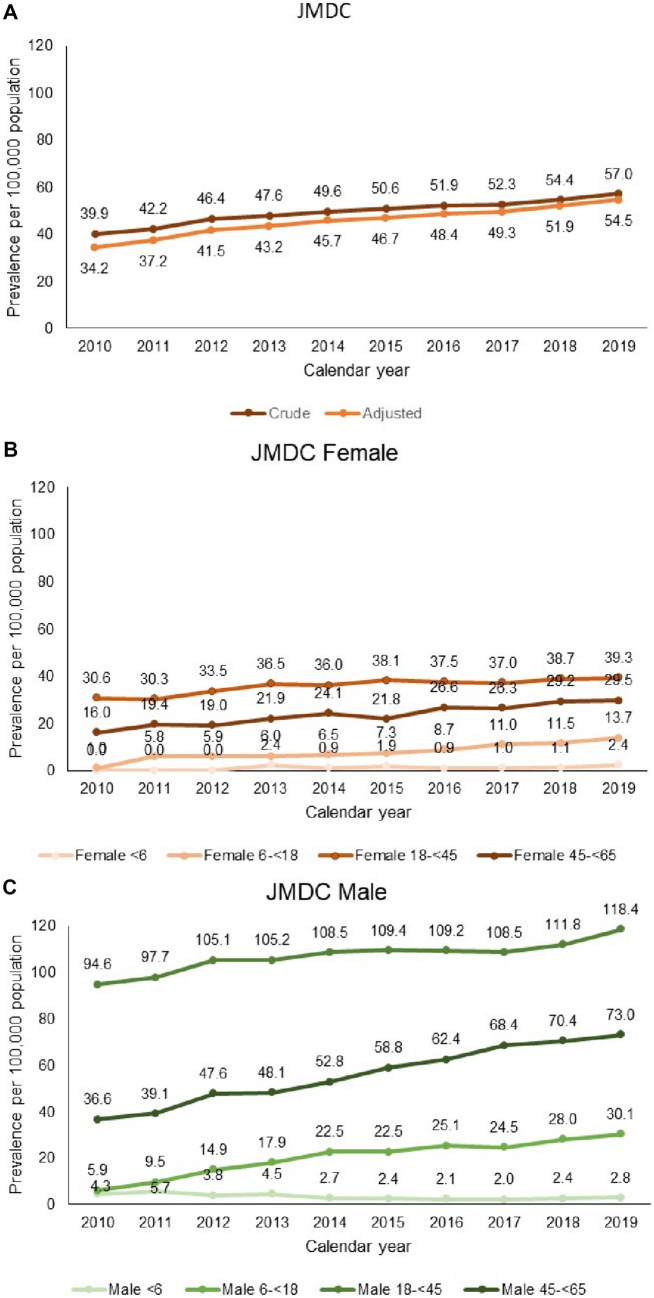
Annual period prevalence (per 100,000 population) of CD in the JMDC (Japan). **A** Overall—crude and adjusted; **B** by age group in females; **C** by age group in males. Data are tabulated in Table S1

**Fig. 3:**
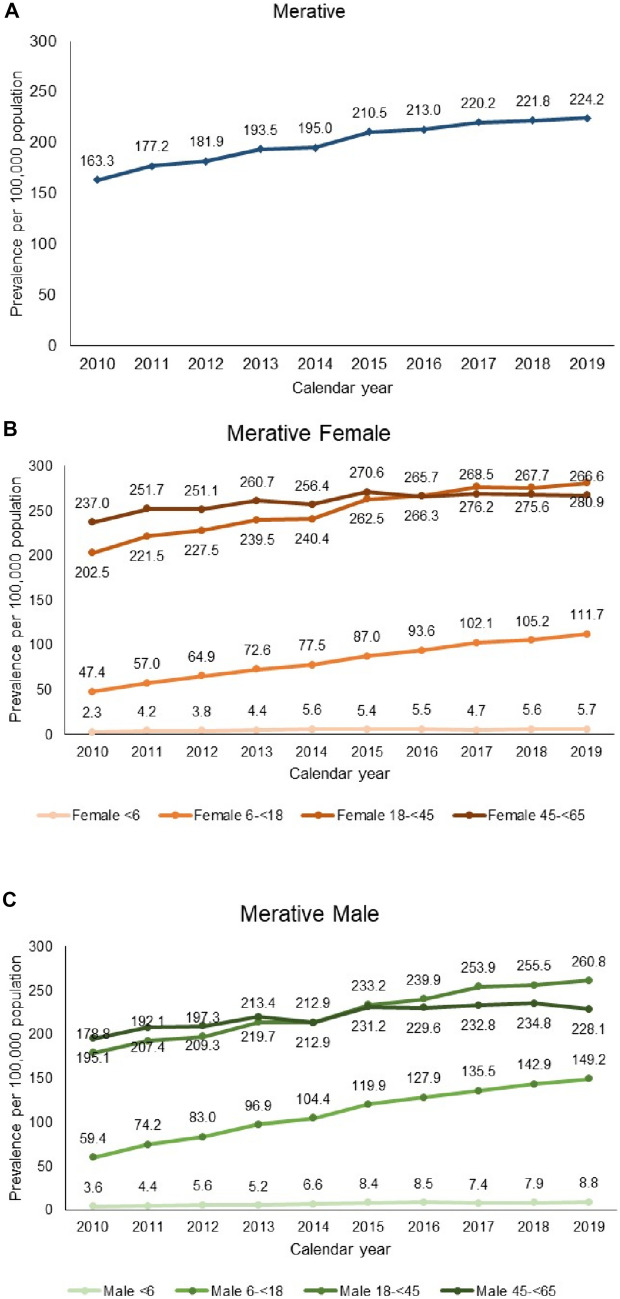
Annual period prevalence (per 100,000 population) of CD in Merative (US). **A** Overall; **B** by age group in females; **C** by age group in males. Data are tabulated in Table S2

The crude period prevalence rates of CD in the JMDC increased annually over the study period from 39.9 per 100,000 population in 2010 to 57.0 per 100,000 population in 2019 (Fig. 2A). Similarly, period prevalence rates of CD in Merative increased annually from 163.3 per 100,000 population in 2010 to 224.2 per 100,000 population in 2019 (Fig. 3A). When adjusted for age and sex using the Merative population as the standard population, the period prevalence of CD in the JMDC still increased annually from 34.2 per 100,000 population in 2010 to 54.5 per 100,000 population in 2019 (Fig. 2A).

### Category-specific prevalence in the JMDC

For all age groups, the prevalence of CD was higher in males than in females in JMDC over the entire study period (Fig. 2B and C). The highest prevalence rates were observed in the 18- to < 45-year age group in both males and females but was approximately threefold higher in males (118.4 per 100,000 population in males vs. 39.3 per 100,000 population in females in 2019). The prevalence of CD increased through the study years in all age categories other than those aged < 6 years.

### Category-specific prevalence in Merative

The prevalence of CD was higher in females than males in the 18- to < 45-year age groups and 45- to < 65-year age groups in Merative, but lower in females than males for age groups < 18 years (Fig. 3B and C). For both sexes, the prevalence of CD increased over the study period in all age categories other than the < 6-year age group.

The highest prevalence of CD was observed in the oldest population aged 45 to < 65 years in males and females until 2014 in males, and 2016 in females. Thereafter the highest prevalence rates were observed in 18-year-olds to < 45-year-olds in both males and females.

### Incidence of CD

**Fig. 4:**
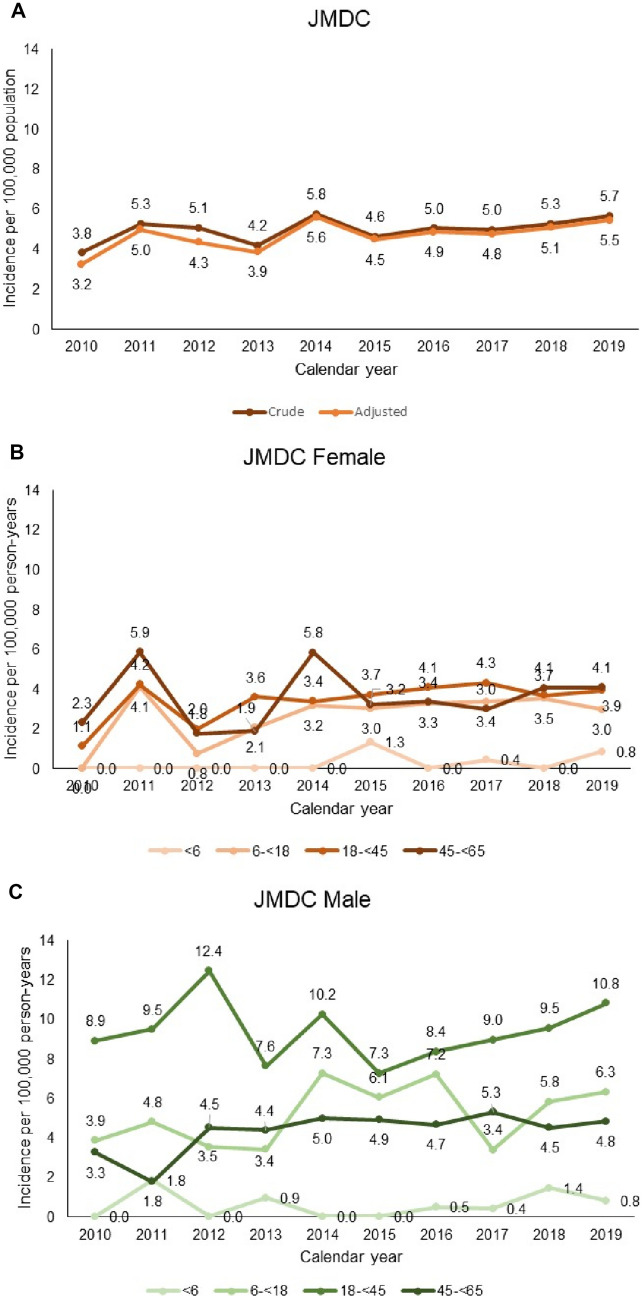
Annual incidence rates (per 100,000 person-years) of CD in the JMDC (Japan). **A** Overall; **B** age group in females; **C** by age group in males. Data are tabulated in Table S3

**Fig. 5:**
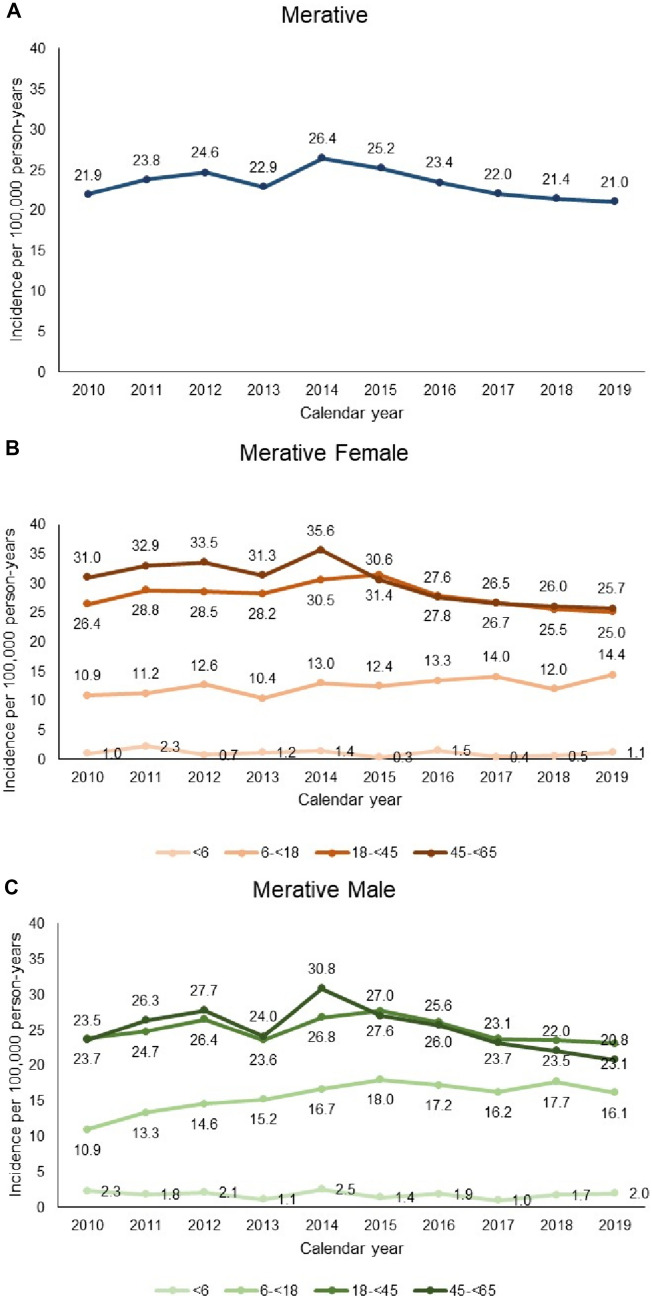
Annual incidence rates (per 100,000 person-years) of CD in Merative (US) database. **A** Overall; **B** age group in females; **C** by age group in males. Data are tabulated in Table S4

The crude period incidence of CD in the JMDC remained largely stable over the study period, ranging from 3.8 to 5.8 per 100,000 person-years between 2010 and 2019 (Fig. 4A). When adjusted for age and sex with the Merative population as the standard population, the period incidence of CD in the JMDC remined stable (3.2 to 5.6 per 100,000 person-years) (Fig. 4A). The incidence rate of CD was higher in Merative than in JMDC but the period incidence also remained stable over the study period, ranging from 21.0 to 26.4 per 100,000 person-years (Fig. 5A).

### Category-specific incidence in the JMDC

The incidence of CD tended to be higher in males than in females in JMDC over the entire study period, with the highest incidence rates observed in 18-year-old to < 45-year-old males (Fig. 4B and C). In females, incidence rates were similar in age groups between 6 and 65 years. The incidence of CD remained stable through the study years in all age group categories in females and males.

### Category-specific incidence in Merative

The incidence of CD was higher in the 18 to < 45 and 45 to < 65 age group categories in both males and females, followed by 6-year-olds to < 18-year-olds (Fig. 5B and C). There was no consistent evidence of any change in incidence over time in any age-group category, or in males or females over the study period.

## Discussion

We used two nationwide claims databases covering both employees and their dependents to evaluate temporal trends in the prevalence and incidence rates of CD in Japan and the US, two developed countries with advanced healthcare systems and with different ethnic composition.

The epidemiology of CD differed in each country. In 2019, the prevalence of CD was more than fourfold higher in the US than in Japan (224.2 vs. 54.5 [standardized rate] per 100,000 population). Patients with CD in Japan were of younger age than patients with CD in the US at each time point. Approximately 70% of CD cases were male in Japan vs. a small preponderance of females over males with CD in the US. The prevalence of CD increased substantially over the 10-year study period in both countries. In Japan, the increase was most marked in 45 year-olds to < 65-year-olds and 6 year-olds to < 18-year-olds in both males and females; however, the highest prevalence rates for both male and female were in the 18–45-year-old groups. In the US, the increase was most marked in 6 year-olds to < 18-year-olds of both sexes with the highest prevalence rates in older females and males.

In 2019, the incidence rate of CD was also almost fourfold higher in the US than in Japan (21.0 vs. 5.5 [standardized rate] per 100,000 person-years). However, in both countries, incidence rates remained relatively stable in both sexes and in all age group categories.

Our results are generally consistent with other studies that have used national level data sources in Japan and the US. A national survey of hospitals in Japan estimated an annual prevalence rate of CD of 55.6 per 100,000 population (95% CI 44.6–66.6) in 2014 [9], which is similar to our 2014 estimate of 49.6 per 100,000 population. The male-to-female ratio in CD was 2.40, which is very close to our estimate (70/30 = 2.3). A nation-wide inception cohort registry study from 2018 to 2020 reported that the peak age of diagnosis was in 16–45-year-olds, with approximately 70% of cases in males [19], which is also consistent with our findings. The strikingly higher incidence of IBD in males than in females in Asian countries has been reported previously [20]. To date, there is no conclusive explanation for this difference but sex differences in environmental and social risk profiles, underlying genetic predisposition, immune dysregulation, and microbial dysbiosis have been proposed [20]. Additionally, the male preponderance in our study may be due to the gender distributions of the study cohorts, which were obtained from a claims database covering employees and their dependents; although, the gender distribution of the whole database is not known, and we are unable to test this hypothesis.

The 2011 prevalence of CD in a study conducted on Olmsted County, Minnesota in the US was 246.7 per 100,000 persons (95% CI 221.7–271.8) [11], which is higher than our 2011 estimate of 177.2 per 100,000 population. However, interrogation of two national databases in the US (Optum Clinformatics DataMart and Truven Health MarketScan Research databases) found that the standardized prevalence (pooled databases and adjusted to the US population for age, sex, and geographic region) of CD in 2016 was 45.9 per 100,000 (95% CI 44.1–47.47) children aged 2–17 years. and 197.7 (95% CI 195.8–199.6) per 100,000 adults from 18 years of age [10]. Together, these are close to our own 2016 estimate of 213.0 per 100,000 population.

Our study provides contemporary national-level information on the burden of CD in Japan and the US. The study shows that the incidence of CD in Japan and US, although very different, remained stable over the 10-year period between 2010 and 2019. Both countries recorded substantial and continuous increases in disease prevalence consistent with improved survival in CD [21–23]. CD represents a significant burden to healthcare systems, incurs high direct and indirect costs, negatively impacts quality of life of patients, and has wider societal impacts on employment and productivity [24, 25]. The total economic burden of CD in the US in 2006 was estimated to be 10.9–15.5 billion dollars [26]; although, this figure is now likely to be substantially higher in view of the rising prevalence observed in intervening years.

Strengths of our study include the use of nationwide databases with similar member populations in two different countries—one Western, one Asia, the large sample size, and the 10-year study period that allowed us to visualize temporal trends. Standardization of the JMDC to the Merative population allowed direct comparison of the two populations. To our knowledge, this is the first study to report sex and age category–specific prevalence and incidence rates in Japan, and one of few national-level studies that have investigated IBD epidemiology in the US.

Potential limitations of the study include the lack of data in adults aged 65 years and older due to the rules guiding membership in Merative, and the potential for selection bias given that persons who are unable to work due to illness are not captured in the employment databases. Nevertheless, CD is typically diagnosed during adult life and the population of patients with CD in the JMDC and Merative databases may be considered representative of the population of adults with CD in their respective countries. Although we employed a well-established approach for case identification, misclassification leading to over- or underestimation of CD diagnoses remains a potential issue.

In conclusion, the incidence of CD in Japan and the US appears to remain relatively stable over period from 2010 to 2019, whereas prevalence rates in age groups > 6 years increased in both countries. Despite these apparent similarities, prevalence rates, incidence rates, age, and sex distribution of CD differ substantially between Japan and the US. Research to understand the basis of these differences could help to identify at-risk groups in each country, and guide implementation of preventive measures.

## Supplementary Information

Below is the link to the electronic supplementary material.

Supplementary file1 (DOCX 79 KB)

## Data Availability

The data underlying this article were provided by the JMDC and Merative under license. Data will be shared on request to the corresponding author with permission of JMDC and/or Merative.
