# Supplementary material for: Trends in the prevalence and incidence of Crohn’s disease in Japan and the United States
Source: Int J Colorectal Dis. 2024 Apr 27;39(1):61. doi: 10.1007/s00384-024-04636-5 (PMC11055788; doi:10.1007/s00384-024-04636-5)
Supplement: Supplementary file 1 — Supplementary file1 (DOCX 79 KB) [file 384_2024_4636_MOESM1_ESM.docx]

# **Trends in the Prevalence and Incidence of Crohn’s Disease in Japan and the United States**

**SUPPLEMENT**

**Table S1** Period prevalence of Crohn’s disease (CD) for each calendar year, overall and by age and sex – Japan

| **Age (years)** |  | **2010** | **2011** | **2012** | **2013** | **2014** | **2015** | **2016** | **2017** | **2018** | **2019** |
| --- | --- | --- | --- | --- | --- | --- | --- | --- | --- | --- | --- |
| All <6 | Total N patients | 44474 | 68688 | 103434 | 174414 | 217595 | 326255 | 455985 | 591901 | 727340 | 697483 |
|  | N with CD | 1 | 2 | 2 | 6 | 4 | 7 | 7 | 9 | 13 | 18 |
|  | per 100,000 | 2.25 | 2.91 | 1.93 | 3.44 | 1.84 | 2.15 | 1.54 | 1.52 | 1.79 | 2.58 |
| All 6-<18 | Total N patients | 197667 | 245821 | 314212 | 480059 | 501973 | 701580 | 900329 | 1058461 | 1180113 | 1123218 |
|  | N with CD | 7 | 19 | 33 | 58 | 74 | 106 | 154 | 190 | 235 | 248 |
|  | per 100,000 | 3.5 | 7.7 | 10.5 | 12.1 | 14.7 | 15.1 | 17.1 | 18.0 | 19.9 | 22.1 |
| All 18<45 | Total N patients | 446,175 | 574,141 | 741,901 | 1,163,702 | 1,252,708 | 1,793,491 | 2,434,143 | 2,994,276 | 3,420,115 | 3,143,667 |
|  | N with CD | 301 | 396 | 555 | 886 | 975 | 1,417 | 1,895 | 2,288 | 2,707 | 2,629 |
|  | per 100,000 | 67 | 69 | 75 | 76 | 78 | 79 | 78 | 76 | 79 | 84 |
| All 45<65 | Total N patients | 288027 | 372363 | 494986 | 784166 | 816323 | 1259986 | 1671305 | 1991383 | 2205426 | 2063367 |
|  | N with CD | 81 | 115 | 177 | 290 | 331 | 535 | 776 | 983 | 1144 | 1109 |
|  | per 100,000 | 28 | 31 | 36 | 37 | 41 | 42 | 46 | 49 | 52 | 54 |
| **Males** | All males | 550632 | 708497 | 932723 | 1457899 | 1560038 | 2260758 | 2989374 | 3585768 | 4083910 | 3843998 |
|  | All males with CD | 312 | 421 | 614 | 971 | 1092 | 1625 | 2193 | 2675 | 3148 | 3108 |
|  | per 100,000 | 57 | 59 | 66 | 67 | 70 | 72 | 73 | 75 | 77 | 81 |
| <6 | Total N patients | 23034 | 35354 | 53298 | 89843 | 112075 | 167781 | 234539 | 304084 | 373399 | 358029 |
|  | N with CD | 1 | 2 | 2 | 4 | 3 | 4 | 5 | 6 | 9 | 10 |
|  | per 100,000 | 4.34 | 5.66 | 3.75 | 4.45 | 2.68 | 2.38 | 2.13 | 1.97 | 2.41 | 2.79 |
| 6-<18 | Total N patients | 101416 | 126046 | 161164 | 246207 | 257367 | 359810 | 461555 | 542153 | 604241 | 574905 |
|  | N with CD | 6 | 12 | 24 | 44 | 58 | 81 | 116 | 133 | 169 | 173 |
|  | per 100,000 | 5.9 | 9.5 | 14.9 | 17.9 | 22.5 | 22.5 | 25.1 | 24.5 | 28.0 | 30.1 |
| 18<45 | Total N patients | 256927 | 329620 | 428230 | 670953 | 722487 | 1029271 | 1368816 | 1649170 | 1891099 | 1762406 |
|  | N with CD | 243 | 322 | 450 | 706 | 784 | 1126 | 1495 | 1790 | 2115 | 2086 |
|  | per 100,000 | 95 | 98 | 105 | 105 | 109 | 109 | 109 | 109 | 112 | 118 |
| 45<65 | Total N patients | 169255 | 217477 | 290031 | 450896 | 468109 | 703896 | 924464 | 1090361 | 1215171 | 1148658 |
|  | N with CD | 62 | 85 | 138 | 217 | 247 | 414 | 577 | 746 | 855 | 839 |
|  | per 100,000 | 37 | 39 | 48 | 48 | 53 | 59 | 62 | 68 | 70 | 73 |
| **Females** | All females | 425,711 | 552,516 | 721,810 | 1,144,442 | 1,228,561 | 1,820,554 | 2,472,388 | 3,050,253 | 3,449,084 | 3,183,737 |
|  | All females with CD | 78 | 111 | 153 | 269 | 292 | 440 | 639 | 795 | 951 | 896 |
|  | per 100,000 | 18 | 20 | 21 | 24 | 24 | 24 | 26 | 26 | 28 | 28 |
| <6 | Total N patients | 21440 | 33334 | 50136 | 84571 | 105520 | 158474 | 221446 | 287817 | 353941 | 339454 |
|  | N with CD | 0 | 0 | 0 | 2 | 1 | 3 | 2 | 3 | 4 | 8 |
|  | per 100,000 | 0.0 | 0.0 | 0.0 | 2.4 | 0.9 | 1.9 | 0.9 | 1.0 | 1.1 | 2.4 |
| 6-<18 | Total N patients | 96251 | 119775 | 153048 | 233852 | 244606 | 341770 | 438774 | 516308 | 575872 | 548313 |
|  | N with CD | 1 | 7 | 9 | 14 | 16 | 25 | 38 | 57 | 66 | 75 |
|  | per 100,000 | 18 | 24 | 25 | 32 | 36 | 34 | 36 | 39 | 41 | 44 |
| 18<45 | Total N patients | 189,248 | 244,521 | 313,671 | 492,749 | 530,221 | 764,220 | 1,065,327 | 1,345,106 | 1,529,016 | 1,381,261 |
|  | N with CD | 58 | 74 | 105 | 180 | 191 | 291 | 400 | 498 | 592 | 543 |
|  | per 100,000 | 31 | 30 | 33 | 37 | 36 | 38 | 38 | 37 | 39 | 39 |
| 45<65 | Total N patients | 118772 | 154886 | 204955 | 333270 | 348214 | 556090 | 746841 | 901022 | 990255 | 914709 |
|  | N with CD | 19 | 30 | 39 | 73 | 84 | 121 | 199 | 237 | 289 | 270 |
|  | per 100,000 | 16 | 19 | 19 | 22 | 24 | 22 | 27 | 26 | 29 | 30 |

**Table S2** Period prevalence of Crohn’s disease (CD) for each calendar year, overall and by age and sex – Merative

| **Age (years)** |  | **2010** | **2011** | **2012** | **2013** | **2014** | **2015** | **2016** | **2017** | **2018** | **2019** |
| --- | --- | --- | --- | --- | --- | --- | --- | --- | --- | --- | --- |
| <6 | Total N patients | 1967985 | 2236339 | 2249357 | 1860419 | 1866791 | 1442719 | 1421745 | 1318430 | 1312043 | 1246931 |
|  | N with CD | 58 | 97 | 107 | 90 | 114 | 100 | 100 | 80 | 89 | 91 |
|  | per 100,000 | 2.95 | 4.34 | 4.76 | 4.84 | 6.11 | 6.93 | 7.03 | 6.07 | 6.78 | 7.30 |
| All 6-<18 | Total N patients | 5176770 | 5846968 | 6092463 | 4986919 | 5133916 | 3898029 | 3873997 | 3549835 | 3500352 | 3373635 |
|  | N with CD | 2770 | 3844 | 4519 | 4239 | 4685 | 4046 | 4303 | 4229 | 4355 | 4414 |
|  | per 100,000 | 54 | 66 | 74 | 85 | 91 | 104 | 111 | 119 | 124 | 131 |
| All 18<45 | Total N patients | 11567741 | 13651191 | 14555427 | 12109922 | 12788003 | 9726893 | 9746290 | 8968628 | 9071162 | 8843100 |
|  | N with CD | 22117 | 28299 | 30980 | 27478 | 29049 | 24163 | 24720 | 23810 | 24117 | 23977 |
|  | per 100,000 | 191 | 207 | 213 | 227 | 227 | 248 | 254 | 265 | 266 | 271 |
| All 45<65 | Total N patients | 10,373,662 | 11710272 | 12198069 | 10160886 | 10,759,408 | 8151377 | 8165742 | 7456674 | 7365622 | 7049084 |
|  | N with CD | 22542 | 27019 | 28220 | 24540 | 25729 | 20562 | 20319 | 18769 | 18568 | 17502 |
|  | per 100,000 | 217 | 231 | 231 | 242 | 239 | 252 | 249 | 252 | 252 | 248 |
| **Males** | All males | 14032511 | 16258290 | 17076206 | 14096406 | 14879080 | 11197338 | 11201162 | 10315613 | 10346008 | 10001899 |
|  | All males with CD | 20,958 | 26,408 | 28,621 | 25,429 | 26,861 | 22,117 | 22,583 | 21,645 | 22,037 | 21,462 |
|  | per 100,000 | 149 | 162 | 168 | 180 | 181 | 198 | 202 | 210 | 213 | 215 |
| <6 | Total N patients | 1008224 | 1147058 | 1153372 | 954243 | 956532 | 739568 | 727874 | 674437 | 671041 | 637452 |
|  | N with CD | 36 | 51 | 65 | 50 | 63 | 62 | 62 | 50 | 53 | 56 |
|  | per 100,000 | 3.57 | 4.45 | 5.64 | 5.24 | 6.59 | 8.38 | 8.52 | 7.41 | 7.90 | 8.78 |
| 6-<18 | Total N patients | 2643056 | 2983504 | 3108209 | 2543794 | 2619585 | 1989231 | 1976087 | 1810834 | 1785038 | 1721075 |
|  | N with CD | 1569 | 2213 | 2581 | 2465 | 2736 | 2385 | 2527 | 2453 | 2551 | 2568 |
|  | per 100,000 | 59 | 74 | 83 | 97 | 104 | 120 | 128 | 135 | 143 | 149 |
| 18<45 | Total N patients | 5505025 | 6593577 | 7057098 | 5841142 | 6149918 | 4674108 | 4683342 | 4323020 | 4393023 | 4289228 |
|  | N with CD | 9841 | 12668 | 13924 | 12463 | 13092 | 10898 | 11236 | 10977 | 11223 | 11186 |
|  | per 100,000 | 179 | 192 | 197 | 213 | 213 | 233 | 240 | 254 | 255 | 261 |
| 45<65 | Total N patients | 4876206 | 5534151 | 5757527 | 4757227 | 5153045 | 3794431 | 3813859 | 3507322 | 3496906 | 3354144 |
|  | N with CD | 9,512 | 11,476 | 12,051 | 10,451 | 10,970 | 8,772 | 8,758 | 8,165 | 8,210 | 7,652 |
|  | per 100,000 | 195 | 207 | 209 | 220 | 213 | 231 | 230 | 233 | 235 | 228 |
| **Females** | All females | 15,053,647 | 17,186,480 | 18,019,110 | 15,021,740 | 15,669,038 | 12,021,680 | 12,006,612 | 10,977,954 | 10,903,171 | 10,510,851 |
|  | All females with CD | 26,529 | 32,851 | 35,205 | 30,918 | 32,716 | 26,754 | 26,859 | 25,243 | 25,092 | 24,522 |
|  | per 100,000 | 176 | 191 | 195 | 206 | 209 | 223 | 224 | 230 | 230 | 233 |
| <6 | Total N patients | 959761 | 1089281 | 1095985 | 906176 | 910259 | 703151 | 693871 | 643993 | 641002 | 609479 |
|  | N with CD | 22 | 46 | 42 | 40 | 51 | 38 | 38 | 30 | 36 | 35 |
|  | per 100,000 | 2.3 | 4.2 | 3.8 | 4.4 | 5.6 | 5.4 | 5.5 | 4.7 | 5.6 | 5.7 |
| 6-<18 | Total N patients | 2533714 | 2863464 | 2984254 | 2443125 | 2514331 | 1908798 | 1897910 | 1739001 | 1715314 | 1652560 |
|  | N with CD | 1201 | 1631 | 1938 | 1774 | 1949 | 1661 | 1776 | 1776 | 1804 | 1846 |
|  | per 100,000 | 47 | 57 | 65 | 73 | 78 | 87 | 94 | 102 | 105 | 112 |
| 18<45 | Total N patients | 6062716 | 7057614 | 7498329 | 6268780 | 6638085 | 5052785 | 5062948 | 4645608 | 4678139 | 4553872 |
|  | N with CD | 12276 | 15631 | 17056 | 15015 | 15957 | 13265 | 13484 | 12833 | 12894 | 12791 |
|  | per 100,000 | 202 | 221 | 227 | 240 | 240 | 263 | 266 | 276 | 276 | 281 |
| 45<65 | Total N patients | 5497456 | 6176121 | 6440542 | 5403659 | 5,756,439 | 4356946 | 4351883 | 3949352 | 3868716 | 3694940 |
|  | N with CD | 13,030 | 15,543 | 16,169 | 14,089 | 14,759 | 11,790 | 11,561 | 10,604 | 10,358 | 9,850 |
|  | per 100,000 | 237 | 252 | 251 | 261 | 256 | 271 | 266 | 268 | 268 | 267 |

**Table S3** Period incidence of Crohn’s disease (CD) per 100,000 person-years (py) for each calendar year, overall and by age and sex – Japan

| **Age (years)** | | **2010** | **2011** | **2012** | **2013** | **2014** | **2015** | **2016** | **2017** | **2018** | **2019** |
| --- | --- | --- | --- | --- | --- | --- | --- | --- | --- | --- | --- |
| <6 | pys of follow-up | 79414 | 105718 | 139198 | 206392 | 232635 | 311020 | 407536 | 484960 | 541275 | 490511 |
|  | N with CD | 0 | 1 | 0 | 1 | 0 | 2 | 1 | 2 | 4 | 4 |
|  | per 100,000 py | 0.00 | 0.95 | 0.00 | 0.48 | 0.00 | 0.64 | 0.25 | 0.41 | 0.74 | 0.82 |
| 6-<18 | Total N patients | 149880 | 201494 | 273283 | 399179 | 455199 | 611364 | 810166 | 980105 | 1104637 | 1110077 |
|  | N with CD | 3 | 9 | 6 | 11 | 24 | 28 | 43 | 33 | 52 | 52 |
|  | per 100,000 py | 2.0 | 4.5 | 2.2 | 2.8 | 5.3 | 4.6 | 5.3 | 3.4 | 4.7 | 4.7 |
| 18<45 | Total N patients | 424886 | 562824 | 729829 | 1060031 | 1193784 | 1597141 | 2164307 | 2670056 | 2992484 | 2847789 |
|  | N with CD | 24 | 41 | 59 | 63 | 88 | 92 | 141 | 184 | 208 | 222 |
|  | per 100,000 py | 5.6 | 7.3 | 8.1 | 5.9 | 7.4 | 5.8 | 6.5 | 6.9 | 7.0 | 7.8 |
| 45<65 | Total N patients | 209364 | 287836 | 415371 | 630094 | 731524 | 1077583 | 1493614 | 1872374 | 2161134 | 2224014 |
|  | N with CD | 6 | 10 | 14 | 21 | 39 | 45 | 61 | 80 | 93 | 100 |
|  | per 100,000 py | 2.9 | 3.5 | 3.4 | 3.3 | 5.3 | 4.2 | 4.1 | 4.3 | 4.3 | 4.5 |
| **Males** | All males | 488142.46 | 653459.21 | 883088.21 | 1293304.97 | ########## | 2007764.17 | 2694238.09 | ########## | 3708628.85 | 3664570.82 |
|  | All males with CD | 29 | 40 | 69 | 71 | 109 | 116 | 173 | 206 | 250 | 271 |
|  | per 100,000 py | 5.9 | 6.1 | 7.8 | 5.5 | 7.4 | 5.8 | 6.4 | 6.3 | 6.7 | 7.4 |
| <6 | Total N patients | 40854.63929 | 54337.782 | 71508.46 | 106171.4032 | 119639.58 | 159919.2 | 209340.09 | 249240.86 | 277980.63 | 251533.53 |
|  | N with CD | 0 | 1 | 0 | 1 | 0 | 0 | 1 | 1 | 4 | 2 |
|  | per 100,000 py | 0.00 | 1.84 | 0.00 | 0.94 | 0.00 | 0.00 | 0.48 | 0.40 | 1.44 | 0.80 |
| 6-<18 | Total N patients | 77577.84257 | 104080.9 | 140920.94 | 205418.2478 | 234170.08 | 314019.83 | 415547.34 | 502494.74 | 566112.45 | 569547.44 |
|  | N with CD | 3 | 5 | 5 | 7 | 17 | 19 | 30 | 17 | 33 | 36 |
|  | per 100,000 py | 3.9 | 4.8 | 3.5 | 3.4 | 7.3 | 6.1 | 7.2 | 3.4 | 5.8 | 6.3 |
| 18<45 | Total N patients | 246961.00 | 326729.42 | 425920.48 | 615718.22 | 693070.16 | 923057.03 | 1231724.86 | ########## | 1665794.07 | 1601938.07 |
|  | N with CD | 22 | 31 | 53 | 47 | 71 | 67 | 103 | 133 | 159 | 173 |
|  | per 100,000 py | 8.9 | 9.5 | 12.4 | 7.6 | 10.2 | 7.3 | 8.4 | 9.0 | 9.5 | 10.8 |
| 45<65 | Total N patients | 122748.98 | 168311.11 | 244738.33 | 365997.10 | 422912.39 | 610768.11 | 837625.79 | ########## | 1198741.71 | 1241551.78 |
|  | N with CD | 4 | 3 | 11 | 16 | 21 | 30 | 39 | 55 | 54 | 60 |
|  | per 100,000 py | 3.3 | 1.8 | 4.5 | 4.4 | 5.0 | 4.9 | 4.7 | 5.3 | 4.5 | 4.8 |
| **Females** | All females | 375,401 | 504,413 | 674,592 | 1,002,391 | 1,143,351 | 1,589,343 | 2,181,384 | 2,733,462 | 3,090,900 | 3,007,820 |
|  | All females with CD | 4 | 21 | 10 | 25 | 42 | 51 | 73 | 93 | 107 | 107 |
|  | per 100,000 py | 1.1 | 4.2 | 1.5 | 2.5 | 3.7 | 3.2 | 3.3 | 3.4 | 3.5 | 3.6 |
| <6 | Total N patients | 38559.36482 | 51380.301 | 67689.309 | 100220.4381 | 112995.64 | 151100.43 | 198195.44 | 235719.64 | 263293.91 | 238977.91 |
|  | N with CD | 0 | 0 | 0 | 0 | 0 | 2 | 0 | 1 | 0 | 2 |
|  | per 100,000 py | 0.00 | 0.00 | 0.00 | 0.00 | 0.00 | 1.32 | 0.00 | 0.42 | 0.00 | 0.84 |
| 6-<18 | Total N patients | 72302.10815 | 97413.363 | 132361.98 | 193760.3285 | 221029.4 | 297344.05 | 394618.83 | 477610.52 | 538524.14 | 540529.28 |
|  | N with CD | 0 | 4 | 1 | 4 | 7 | 9 | 13 | 16 | 19 | 16 |
|  | per 100,000 py | 0.00 | 4.11 | 0.76 | 2.06 | 3.17 | 3.03 | 3.29 | 3.35 | 3.53 | 2.96 |
| 18<45 | Total N patients | 177924.501 | 236094.33 | 303908.08 | 444313.232 | 500713.72 | 674083.75 | 932581.77 | 1185335.3 | 1326689.7 | 1245850.8 |
|  | N with CD | 2 | 10 | 6 | 16 | 17 | 25 | 38 | 51 | 49 | 49 |
|  | per 100,000 py | 1.12 | 4.24 | 1.97 | 3.60 | 3.40 | 3.71 | 4.07 | 4.30 | 3.69 | 3.93 |
| 45<65 | Total N patients | 86,615 | 119,525 | 170,632 | 264,097 | 308,612 | 466,815 | 655,988 | 834,797 | 962,392 | 982,462 |
|  | N with CD | 2 | 7 | 3 | 5 | 18 | 15 | 22 | 25 | 39 | 40 |
|  | per 100,000 py | 2.31 | 5.86 | 1.76 | 1.89 | 5.83 | 3.21 | 3.35 | 2.99 | 4.05 | 4.07 |

**Table S4** Period incidence of Crohn’s disease (CD)per 100,000 person-years (py) for each calendar year, overall and by age and sex – Merative

| **Age (years)** | | **2010** | **2011** | **2012** | **2013** | **2014** | **2015** | **2016** | **2017** | **2018** | **2019** |
| --- | --- | --- | --- | --- | --- | --- | --- | --- | --- | --- | --- |
| <6 | pys of follow-up | 1693795.63 | 1902071.7 | 1958752.46 | 1586210.33 | 1471338.513 | 1226809.77 | 1237869.05 | 1143469.429 | 1131559.85 | 1085276.339 |
|  | N with CD | 28 | 39 | 28 | 18 | 29 | 11 | 21 | 8 | 13 | 17 |
|  | per 100,000 py | 1.65 | 2.05 | 1.43 | 1.13 | 1.97 | 0.90 | 1.70 | 0.70 | 1.15 | 1.57 |
| 6-<18 | Total N patients | 4763498.73 | 5328321.3 | 5600863 | 4554788.178 | 4299707.912 | 3564683.04 | 3593754.12 | 3287237.029 | 3226240.47 | 3096619.373 |
|  | N with CD | 519 | 653 | 764 | 584 | 639 | 544 | 550 | 498 | 480 | 473 |
|  | per 100,000 py | 10.90 | 12.26 | 13.64 | 12.82 | 14.86 | 15.26 | 15.30 | 15.15 | 14.88 | 15.27 |
| 18<45 | Total N patients | 10,255,945 | 12,057,233 | 12,995,474 | 10,721,460 | 10,275,401 | 8,572,618 | 8,728,088 | 8,016,903 | 8,056,992 | 7,827,122 |
|  | N with CD | 2579 | 3235 | 3574 | 2785 | 2951 | 2536 | 2354 | 2024 | 1976 | 1886 |
|  | per 100,000 py | 25.15 | 26.83 | 27.50 | 25.98 | 28.72 | 29.58 | 26.97 | 25.25 | 24.53 | 24.10 |
| 45<65 | Total N patients | 9,692,469 | 10,850,204 | 11,361,828 | 9,436,840 | 9,020,935 | 7,596,221 | 7,687,850 | 7,004,581 | 6,885,540 | 6,534,614 |
|  | N with CD | 2663 | 3233 | 3497 | 2631 | 3007 | 2195 | 2050 | 1747 | 1661 | 1525 |
|  | per 100,000 py | 27.47 | 29.80 | 30.78 | 27.88 | 33.33 | 28.90 | 26.67 | 24.94 | 24.12 | 23.34 |
| Males | All males | 12723507.95 | 14629458.30 | 15529159.84 | 12721664.69 | 12104200.24 | 10099014.80 | 10251976.70 | 9420822.50 | 9397091.99 | 9040172.87 |
|  | All males with CD | 2528 | 3194 | 3620 | 2659 | 3027 | 2443 | 2353 | 1972 | 1951 | 1799 |
|  | per 100,000 py | 19.87 | 21.83 | 23.31 | 20.90 | 25.01 | 24.19 | 22.95 | 20.93 | 20.76 | 19.90 |
| <6 | Total N patients | 867779.923 | 975258.32 | 1004454.63 | 813438.0534 | 754347.6057 | 628365.473 | 633955.904 | 584979.9398 | 578762.171 | 554643.9151 |
|  | N with CD | 20 | 18 | 21 | 9 | 19 | 9 | 12 | 6 | 10 | 11 |
|  | per 100,000 py | 2.30 | 1.85 | 2.09 | 1.11 | 2.52 | 1.43 | 1.89 | 1.03 | 1.73 | 1.98 |
| 6-<18 | Total N patients | 2432193.39 | 2718526.88 | 2857595.19 | 2323262.14 | 2193914.75 | 1818795.79 | 1833072.96 | 1677033.59 | 1645904.81 | 1579519.24 |
|  | N with CD | 266 | 361 | 417 | 353 | 366 | 327 | 315 | 272 | 291 | 255 |
|  | per 100,000 py | 10.94 | 13.28 | 14.59 | 15.19 | 16.68 | 17.98 | 17.18 | 16.22 | 17.68 | 16.14 |
| 18<45 | Total N patients | 4872875.81 | 5814430.34 | 6304361.23 | 5170775.75 | 4949015.73 | 4118624.90 | 4195975.17 | 3864435.95 | 3903266.44 | 3798115.98 |
|  | N with CD | 1157 | 1438 | 1667 | 1219 | 1325 | 1138 | 1093 | 916 | 916 | 877 |
|  | per 100,000 py | 23.74 | 24.73 | 26.44 | 23.57 | 26.77 | 27.63 | 26.05 | 23.70 | 23.47 | 23.09 |
| 45<65 | Total N patients | 4550658.82 | 5121242.75 | 5362748.80 | 4414188.75 | 4206922.15 | 3533228.63 | 3588972.67 | 3294373.02 | 3269158.57 | 3107893.73 |
|  | N with CD | 1071 | 1349 | 1486 | 1061 | 1295 | 953 | 919 | 762 | 720 | 645 |
|  | per 100,000 py | 23.54 | 26.34 | 27.71 | 24.04 | 30.78 | 26.97 | 25.61 | 23.13 | 22.02 | 20.75 |
| Females | All females | 13682200.15 | 15508371.47 | 16387757.00 | 13577633.19 | 12963181.58 | 10861316.69 | 10995583.91 | 10031367.57 | 9903239.71 | 9503458.72 |
|  | All females with CD | 3261 | 3966 | 4243 | 3359 | 3599 | 2843 | 2622 | 2305 | 2179 | 2102 |
|  | per 100,000 py | 23.83 | 25.57 | 25.89 | 24.74 | 27.76 | 26.18 | 23.85 | 22.98 | 22.00 | 22.12 |
| <6 | Total N patients | 826015.71 | 926813.34 | 954297.837 | 772772.2765 | 716990.9076 | 598444.296 | 603913.15 | 558489.4894 | 552797.678 | 530632.4244 |
|  | N with CD | 8 | 21 | 7 | 9 | 10 | 2 | 9 | 2 | 3 | 6 |
|  | per 100,000 py | 0.97 | 2.27 | 0.73 | 1.16 | 1.39 | 0.33 | 1.49 | 0.36 | 0.54 | 1.13 |
| 6-<18 | Total N patients | 2331305.34 | 2609794.37 | 2743267.81 | 2231526.04 | 2105793.16 | 1745887.25 | 1760681.17 | 1610203.44 | 1580335.66 | 1517100.13 |
|  | N with CD | 253 | 292 | 347 | 231 | 273 | 217 | 235 | 226 | 189 | 218 |
|  | per 100,000 py | 10.85 | 11.19 | 12.65 | 10.35 | 12.96 | 12.43 | 13.35 | 14.04 | 11.96 | 14.37 |
| 18<45 | Total N patients | 5383069.14 | 6242802.19 | 6691112.64 | 5550683.93 | 5326385.08 | 4453992.92 | 4532112.55 | 4152467.12 | 4153725.36 | 4029005.56 |
|  | N with CD | 1422 | 1797 | 1907 | 1566 | 1626 | 1398 | 1261 | 1108 | 1060 | 1009 |
|  | per 100,000 py | 26.42 | 28.79 | 28.50 | 28.21 | 30.53 | 31.39 | 27.82 | 26.68 | 25.52 | 25.04 |
| 45<65 | Total N patients | 5141809.96 | 5728961.56 | 5999078.72 | 5022650.95 | 4814012.43 | 4062992.22 | 4098877.04 | 3710207.53 | 3616381.01 | 3426720.60 |
|  | N with CD | 1592 | 1884 | 2011 | 1570 | 1712 | 1242 | 1131 | 985 | 941 | 880 |
|  | per 100,000 py | 30.96 | 32.89 | 33.52 | 31.26 | 35.56 | 30.57 | 27.59 | 26.55 | 26.02 | 25.68 |
